# The Impact of a Digital Intervention (Happify) on Loneliness During COVID-19: Qualitative Focus Group

**DOI:** 10.2196/26617

**Published:** 2021-02-08

**Authors:** Eliane M Boucher, Emily C McNaughton, Nicole Harake, Julia L Stafford, Acacia C Parks

**Affiliations:** 1 Happify Health New York, NY United States

**Keywords:** loneliness, digital interventions, COVID-19, qualitative research, perspective, impact, intervention, lonely, mental health, e-mental health, digital health, focus group

## Abstract

**Background:**

Loneliness is a growing area of concern, attracting attention as a public health concern due to its association with a variety of psychological and physical health problems. However, interventions targeting loneliness are less common than interventions for other mental health problems, such as depression and anxiety, and existing interventions focus primarily on building social skills and increasing opportunities for social interaction despite research suggesting these techniques are not the most effective. Furthermore, although there is an increasing need for scalable and convenient interventions, digital interventions for loneliness are even less common.

**Objective:**

Using a qualitative approach, we explore how adults (18-64 years of age) who express wanting to be more connected to others experience loneliness and react to a digital mental health intervention targeting loneliness.

**Methods:**

A total of 11 participants were recruited from a pilot randomized controlled trial exploring the impact of a digital mental health intervention, Happify Health, on loneliness among adults aged 18-64 years who indicated wanting to feel more connected to others when signing up for the platform. Participants were invited to participate in a 3-day asynchronous focus group about their experiences with loneliness, with Happify Health, and with social distancing during the COVID-19 pandemic. All 11 participants completed the focus group in May 2020.

**Results:**

Participants’ responses were coded using thematic analysis, which led to identifying five themes, each with separate subthemes, that could be applied across the 3-day focus group: loneliness, relationships, social distancing, skill acquisition, and coping. Overall, we observed variability across participants in terms of the source of their loneliness, their perceptions of their social connections, and their motivation to reduce feelings of loneliness; however, participants commonly referred to negative self-perceptions as a cause or consequence of loneliness. Participants also varied in the extent to which they felt social distancing increased or decreased feelings of loneliness. In regard to the intervention, participants showed evidence of adopting skills they used to address their loneliness, particularly mindfulness and gratitude, and then using these skills to shift toward more active coping strategies following the intervention, including during the COVID-19 pandemic.

**Conclusions:**

The heterogeneity in participants’ experiences with loneliness described during this focus group emphasizes the subjective and complex nature of loneliness. This highlights the importance of developing loneliness interventions that use a variety of strategies, including both direct and indirect strategies for reducing loneliness. However, based on our data, a key component to loneliness interventions is incorporating strategies for addressing underlying negative self-perceptions that stem from, but also contribute to, loneliness. This data also provides preliminary evidence that digital platforms may be an effective tool for disseminating loneliness interventions while providing the added benefit of offering a productive distraction when feeling lonely.

## Introduction

### Background

Loneliness is the feeling that one’s preferred social relations fall short of their actual social relations [1]. In the last decade, loneliness has become a growing area of concern, and with emerging trends suggesting Americans are less socially connected than before, researchers posit loneliness will only become more of an issue over time [2]. In fact, the former 19th US Surgeon General recently argued loneliness is a public health concern [3], labeling problems with loneliness an epidemic [4].

In industrialized countries, researchers estimate that approximately one-third of the population struggle with loneliness, and one-quarter of those will struggle with severe levels of loneliness [5]. In addition, the number of people coping with loneliness may be higher in some regions than in others. For example, although nationally representative surveys of adults 45 years or older living in the United States indicated that about 35% of people are lonely [6], a study of community-dwelling adults in California found that 76% of their sample reported at least moderate levels of loneliness [7]. The reasons for such variability are unclear; however, loneliness appears to be contagious, occurring in clusters and then spreading through social networks [8], which may explain why loneliness may be unusually high in some geographical areas.

However, loneliness is of particular concern because it can then lead to a number of other psychological and physical problems [9]. For example, daily reports of loneliness predict daytime dysfunction [10], and higher levels of chronic loneliness also predict functional limitations [11], increased systolic blood pressure [12], and greater mortality risk [11,13]. Research with cancer survivors suggests loneliness increases the risk for immune dysregulation, leading to higher levels of pain, fatigue, and depression in female breast cancer survivors [14]. Although a lot of research has focused on the bidirectional relationship between loneliness and depression [11,15], loneliness has also been tied to a higher risk of developing severe common mental disorders, including mood disorders, anxiety disorders, and substance use disorders [16], and to elevated risk of developing Alzheimer disease and other forms of dementia, even when controlling for other risk factors [17,18]. In turn, loneliness predicts increased physician visits among older adults [19] and is associated with higher health care costs [20].

### Loneliness During COVID-19

In light of the global coronavirus pandemic, loneliness has become even more of a public health concern. Although the social distancing measures implemented in many countries appear to be effective in slowing the spread of COVID-19, the disease resulting from SARS-CoV-2 [21], researchers have expressed concern that social distancing may result in increased loneliness [22]. Some preliminary research supports these concerns; studies have shown that older adults [23] as well as younger adults (between 18 and 35 years of age) [24] reported elevated levels of loneliness after social distancing measures were implemented. Importantly, although researchers were concerned about the impact of the pandemic on older adults, research suggests that older adults may be more resilient to the negative effects on mental health [25], and younger adults are actually at greater risk for heightened loneliness during the pandemic [26,27]. These heightened levels of loneliness also then place people at greater risk of developing depression or anxiety. In fact, research during the pandemic suggests loneliness may be the strongest predictor of depression and anxiety, even more so than exposure to COVID-19–related situations (eg, self-isolation or knowing someone who had to self-isolate due to COVID-19) and the presence of underlying chronic conditions [26].

### Reducing Loneliness

Given the public health implications of chronic and widespread loneliness, there has been increased interest in developing loneliness interventions and even more so during the COVID-19 pandemic [28,29]. However, there is surprisingly little research on loneliness interventions [28]. This could be due, in part, to the fact that historically many clinicians treated loneliness as a component of depression [30]. Although research supports these are related but distinct constructs [29], interventions focusing specifically on loneliness remain limited.

Existing loneliness interventions tend to focus on increasing the individual’s opportunities for social contact, enhancing social support, developing social skills, or addressing maladaptive thinking [29,31]. There is evidence that these interventions have a small, but significant, effect on loneliness [31]. Interventions including social cognitive training such as cognitive behavioral therapy appear to be more effective than other types of interventions [31], which suggests loneliness may have more to do with maladaptive thinking than a deficit in social connection per se [29]. Nevertheless, most loneliness interventions continue to be based on an intuitive assumption that loneliness can be treated by improving social connection [29] and, consequently, focus primarily on increasing opportunities for social interaction [32].

In recent years, there has been a push to offer more psychological and behavioral interventions digitally [33]. Digital interventions tend to be more scalable than face-to-face interventions and reduce many of the structural barriers that prevent people from seeking treatment [34-36]. The need for digital interventions has become even more apparent during the global COVID-19 pandemic [37], when many mental health professionals have had to pivot to teletherapy [38], and mental health concerns are expected to become even more prevalent [39]. Although a number of digital mental health interventions have been developed [33,40-42], digital loneliness interventions are in their infancy, and most of the studies testing these interventions are small pilot or feasibility studies published in the past 1-2 years [43-46]. Moreover, some of these interventions have been tested with a sample of participants with another comorbid condition such as social anxiety disorder [46] or psychotic disorder [47]. There is, however, some preliminary evidence that internet-based interventions can be effective at reducing loneliness [43,48].

### Understanding the Experience of Loneliness

To date, most loneliness research has been quantitative in nature, exploring the prevalence, correlates, and consequences of loneliness or testing the effectiveness of loneliness interventions. There are comparatively few qualitative studies on the topic of loneliness, particularly studies exploring lonely people’s reactions to loneliness interventions. Given the complex and subjective nature of loneliness [29,49], better understanding people’s experiences with loneliness and with loneliness interventions may be key to developing effective loneliness interventions. Qualitative insights may permit identifying themes in the etiology or source of loneliness and the way they engage with loneliness interventions or why such interventions may not be effective. For example, in one qualitative study of older adults who reported feeling lonely, older adults were more likely to view their loneliness as a complex and private matter, rather than an illness, and thus were unlikely to seek help [50]. However, loneliness appears to have different causes and consequences in early and middle adulthood compared to late adulthood [51], and thus, similar insights from a younger population of adults would be valuable.

### Study Design and Objectives

**Figure 1:**
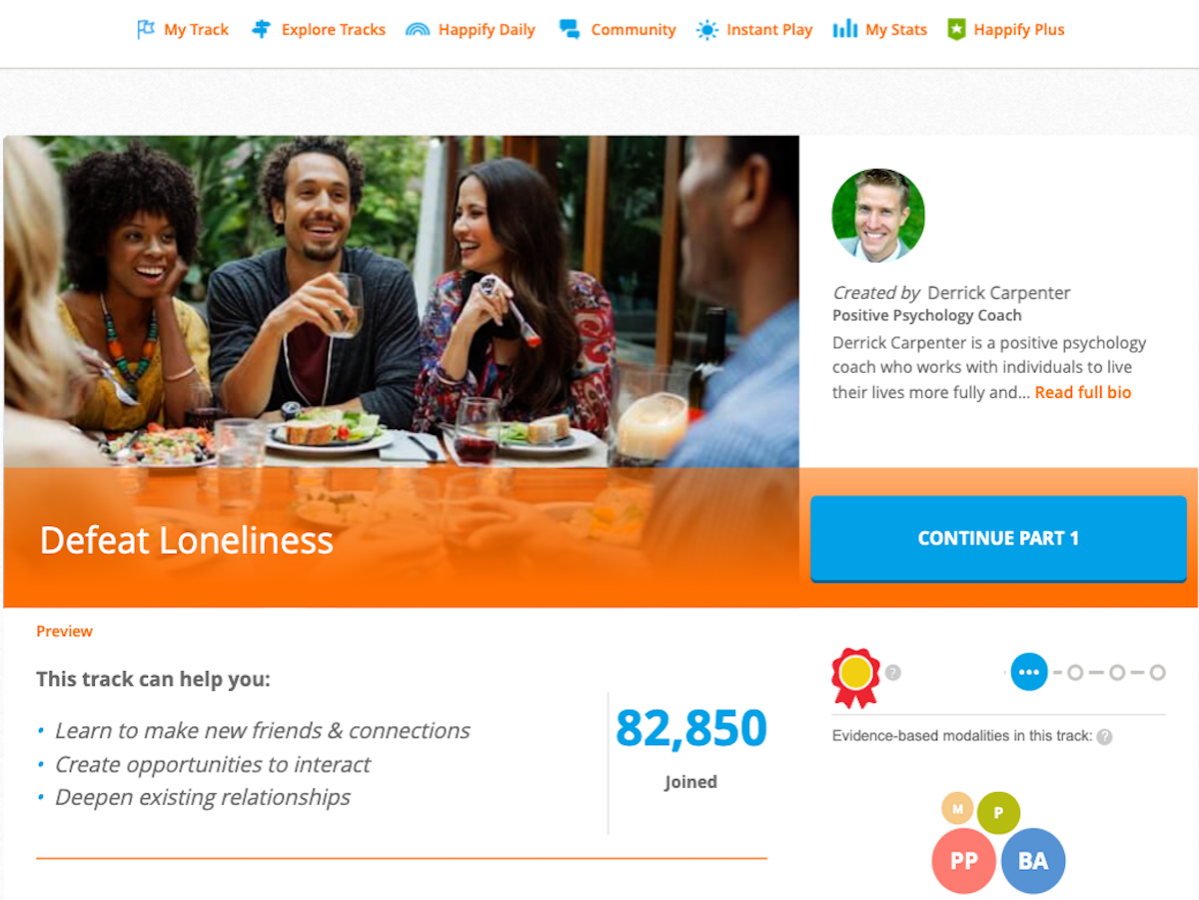
Screenshot of Defeat Loneliness track featured in the pilot randomized controlled trial.

This study draws on a sample of participants who completed a pilot randomized controlled trial (RCT) of the effects of Happify Health among lonely adults between the ages of 18 and 64 years. Happify Health is a digital intervention platform that addresses mental health and its impact on other diseases by drawing on several key theoretical approaches including cognitive behavioral therapy [52], mindfulness-based stress reduction [53], and positive psychology [54]. Users complete gamified versions of evidence-based therapeutic activities (ie, activities with demonstrated effectiveness in at least two different studies with different samples [55]), which are combined into *tracks* that focus on a specific area of concern, like addressing loneliness (ie, “Defeat Loneliness,” see Figure 1).

In the pilot RCT, participants engaged with the Happify Health platform or a psychoeducation control for 8 weeks. We found preliminary evidence that participants who completed more Happify Health activities over 8 weeks had greater improvements in loneliness compared to those who completed fewer activities [56]. To gather additional context, this qualitative study was designed to explore participants’ experiences with loneliness and the Happify Health platform through a multiday virtual, asynchronous focus group. Specific goals included understanding participants’ experiences with loneliness and coping strategies before and after using Happify Health; understanding participants’ experience using Happify Health; and, given that the study was conducted during the COVID-19 pandemic when most of the United States was under some form of a stay-at-home order at the time [57], understanding participants’ experience with social distancing and its relation to perceptions of loneliness.

## Methods

### Participants

Focus group participants were recruited from a pool of individuals who participated in a pilot RCT exploring the impact of Happify Health on loneliness. In the RCT, new users arriving to the Happify Health platform were recruited to participate if they were between the ages of 18 and 64 years, were US residents, and responded “very” to the question “Do you wish you were more connected with others?” when signing up on the platform. Individuals who consented were then randomly assigned to use the Happify Health platform or a corresponding psychoeducation control for 8 weeks (see Parks and Boucher [56] for more discussion of this study). We then invited participants who had been assigned to the active intervention to participate in this focus group if they had completed at least 16 activities on Happify Health (ie, the recommended dosage) and completed the pre- and posttest measures in the pilot study. We emailed 50 participants who met these criteria, and, based on sample sizes in studies with similar focus group designs [58-60], the first 12 participants to indicate interest were accepted into the focus group. Of the 12 individuals, 11 (91.6%) provided informed consent and completed all assigned activities. Of the participants, 9 were female; other demographics were not collected as part of the focus group.

### Procedures

Interested participants were directed to Digsite, a web-based qualitative research tool that enables asynchronous focus groups to provide informed consent and complete a brief introductory activity welcoming them to the group. Participants were given 48 hours to complete these tasks before the first of 3 days of focus group activities were introduced. For each day of the focus group, participants were asked to complete three “activities” on a general topic (see Table 1 or a list of sample questions; full list of focus group questions available in Multimedia Appendix 1). Activities included open-ended discussion prompts and questions as well as a few multiple choice and fill-in-the-blank questions. For the first day, activities focused on learning more about participants’ general perceptions of and experiences with loneliness including coping techniques, while the second day focused on understanding participants’ experiences with Happify Health. On the third day activities focused on understanding participants’ experiences with social distancing due to the COVID-19 pandemic and how this may have impacted their experience of loneliness (the focus group was conducted from April 27 to May 3, 2020; during this time, many of the stay-at-home orders that had been implemented across the United States were still in effect or just recently lifted [61]). Activities were asynchronous, and participants did not interact with each other or the moderator in real time; however, their responses were visible to other focus group participants after they completed each activity, and they were encouraged to read and react to each other’s posts. Similarly, the focus group moderator could pose follow-up questions to participants’ posts.

Participants had 7 days to complete all assigned activities. To encourage participation, participants were compensated US $10 for completing each day of focus group activities and were offered an incentive of US $5 for completing the introductory activity and each of the 3 days of focus group activities within 24 hours of those activities being posted. Consequently, participants could earn up to US $50 for their participation. Participants were also offered free premium subscriptions to Happify Health upon completing the study. All procedures for this study were reviewed and approved by IntegReview, an independent Institutional Review Board.

**Table 1:** Objective and sample questions or prompts by focus group day.

Objective	Sample questions and prompts
Day 1: To understand participants’ general perceptions of and experiences with loneliness including coping techniques	When you first joined Happify, you said that you wished you were more connected to others. Reflecting back, can you tell me about what was going on in your life at the time? In what way(s) were you wishing to be more connected? In your experience, what makes you feel lonely? What do you do when you feel lonely? How do you cope? Can you tell me about the last time you felt lonely and why? When was this? What role does your support system play when you feel lonely?
Day 2: Understand participants’ experiences using Happify Health	What made you want to join Happify? What were you hoping to achieve? When Happify recommended the “Defeat Loneliness” track what did you think? Why? For this task I’d like you to reflect on your journey over the past few months, from the time you started using Happify to today. Tell me about your experience with loneliness during this time. How has Happify played a role in this experience? What did you think about the activities you were asked to complete? Which ones were most and least helpful? Why? Two skills I learned on Happify were ______ and _______.
Day 3: Understanding participants’ experiences with social distancing	How has social distancing changed your day-to-day life in the past month? What is the hardest part of social distancing for you? Why? On a scale of 1 (not at all) to 5 (extremely), when thinking about the next few months, how much is loneliness a concern for you? Please describe why or why not. How are you coping with loneliness during this time? Reflecting on Happify, what skills have you learned that are helping you cope at this time?

### Data Analysis

A verbatim transcript of all focus group activities was exported from Digsite and served as the data corpus for the study. Note that participants’ comments to each other (n=2) and private messages between the moderator and participants (n=23) were also exported; however, upon review, these additional comments did not contribute anything above and beyond participants’ responses to the focus group activities and thus were not included in the thematic analysis. Thematic analysis using an inductive, semantic approach was performed to identify themes and subthemes within the data [62]. To begin, the data were independently reviewed by two members of the study team to create a preliminary set of themes and subthemes for each day of the focus group. These preliminary themes and subthemes were then discussed by both reviewers and a third member of the study team, and overlapping observations were used to create a revised set of themes and subthemes that could be applied across all 3 days of the focus group. The data were then reviewed a second time, applying the revised themes and subthemes. Results were discussed and, as needed, an additional iterative review was conducted until consensus was achieved between the reviewers resulting in a final set of themes and subthemes. Themes were defined as prevalent topics that were observed in responses from the majority of participants. Patterns that were observed less frequently, but were still well-represented within participants’ responses, were classified as subthemes and organized under the appropriate parent theme.

## Results

Coders identified five major themes across responses for the 3-day focus group: loneliness, relationships, social distancing, skill acquisition, and coping. These major themes were further divided into subthemes (see Textbox 1).

**Themes and subthemes extracted from participants’ focus group responses.**

**Loneliness**

- Source of loneliness
- Negative self-appraisal
- Motivation to manage

**Relationships**

- Negative perception of current relationships
- Desire to improve social connection
- Value of social connection

**Social distancing**

- Impact of social distancing on social connection
- General impact of social distancing
- Use of technology

**Skill acquisition**

- Savoring
- Thanks
- Aspire
- Give
- Empathize

**Coping**

- Direct active coping
- Indirect active coping
- Passive coping

### Loneliness

Participants highlighted 3 salient factors that characterized their experiences with loneliness, specifically emphasizing the causes and consequences of loneliness, as well as their motivation to manage their feelings associated with loneliness.

#### Source of Loneliness

When reflecting on their experiences with and perceptions of loneliness, participants commonly described circumstantial reasons for their loneliness; these included life events; relocation; and changes in relationships, employment, and education. Notably, as illustrated by the following participants’ description of loneliness, participants often referred to a constellation of these factors:

I was traveling every week for work and working extremely long hours. A lot of the time I would have colleagues with me, but many times it was just me in a new city. At the same time, I was trying to adjust to my life as an empty nester. I was feeling exhausted from traveling and missing my family, colleagues, and friends.

Similarly, another participant referred to a combination of major life changes in contributing to their loneliness:

At that juncture in my life, my marriage was crumbling, I was experiencing anxiety, and was feeling isolated. I had finished school and had no clue as to how to continue day to day.

In addition to circumstantial triggers for loneliness, participants also mentioned a chronic sense of loneliness. Some participants referred to a history of loneliness since childhood (eg, “Being an only child, I’ve struggled with loneliness from a young age”), whereas others described their loneliness more as a consequence of long-standing mental health issues (eg, “I’ve always struggled with social anxiety and was diagnosed with SAD a few years ago, so I’ve never been good at cultivating and maintaining social relationships.”).

#### Negative Self-appraisal

In describing their loneliness, participants often expressed negative self-appraisals of their perceived self-worth and value to others. Importantly, rather than being another source of loneliness, these negative self-appraisals were typically described as a consequence of their loneliness. In some cases, participants described how lacking social connection led to questions about whether they were to blame for the lack of social connection. For example, as one participant described it:

I began to doubt myself and begin to think, what if it's because my former friends did not perceive me as being of bringing value into their lives and thus that is why, on their end, they did not feel the need to connect with me.

In a similar vein, another participant explained:

Over the years, my loneliness has developed into thoughts of ‘not being good enough to be around,’ ‘being too annoying and clingy,’ and even ‘hating myself so much that I never want to be alone.’

In other cases, it was the lack of social connection that appeared to make participants feel negatively about themselves (eg, “I haven’t done anything today and I’m all by myself - what a waste of space”).

#### Motivation to Manage

The extent to which participants were motivated to address their loneliness varied; this was particularly evident when we asked them to reflect on their reasons for signing up for Happify Health and their reactions when we recommended the *Defeat Loneliness* track as part of the pilot RCT they participated in. Some participants referenced a strong desire to address their feelings of loneliness and cited it as one of their primary reasons for signing up for Happify Health. For example, one participant explained they joined Happify Health because:

I wanted to use evidence-based practices to work on my feelings of loneliness. I wanted to work on feeling more connected.

Another participant described their positive reaction to being recommended the *Defeat Loneliness* track because they currently felt motivated to address their loneliness:

I thought that was the appropriate track to recommended (sic) to me given my feelings of isolation. I felt that I was in a place to finally address this feeling.

Among some participants, however, loneliness was not described as their primary concern and they were drawn to the Happify Health platform because of general concerns with their mental health:

I was feeling a lot of stress and having panic attacks, trouble sleeping, and just generally unhappy. I wanted to have less anxiety and sleep better.

Nevertheless, participants generally expressed positive reactions to the recommended *Defeat Loneliness* track, even when they were not primarily driven to the Happify Health platform because of their loneliness. For instance, the participant who described their reasons for joining Happify Health as they “wanted to have less anxiety and sleep better” described feeling happy when the *Defeat Loneliness* track was recommended:

I was happy because of all things, loneliness is hard to explain to some people. They will say “you have friends or family.” I wanted ways to not feel lonely and also a neutral place to talk about how I felt.

Another participant who described being drawn to Happify to “help me cope with my realities and feel better day to day” described their reaction to the track recommendation as motivating by giving them something concrete to focus on:

I was excited to have a focus. I never identified with the word “loneliness” and then ended up relating to so many of the thoughts and feelings within the track!

### Relationships

Reflecting on their experiences of loneliness often prompted participants to reference their relationships and perceptions of their social network. These comments ranged from expressing dissatisfaction with their current connections and their desire for new or improved connections to emphasizing the importance of social connection.

#### Negative Perception of Current Relationships

Not surprisingly, given that loneliness stems from dissatisfaction with existing social connections [1], several participants described negative perceptions of their current relationships. For example, when asked about the role their support system plays when they are feeling lonely, one participant remarked “I really don’t have one...which sucks.” Another participant described having a support system but feeling different, which contributed to feelings of loneliness:

Usually my friends are super sweet, but talking to them sometimes reminds me of our differences, which then makes me want to isolate.

Participants also often referenced not feeling included by their existing social network. One participant described, “I also noticed that not many people reached out to me,” whereas another described thinking “Why hasn’t anyone called me? Are they doing things without me?” the last time they felt lonely. Such feelings of exclusions were also evident in comments expressing a desire for people in that social network to make greater efforts to include the participant. Some participants noted feeling as though they are always the ones to extend invitations and wanting to be invited for a change (eg, “I did wish that people would approach me- ask me to sit next to them and hang out after class- I was just always the one asking and finding myself rejected by others.”), whereas others referred to a sense of feeling on the outside looking in:

I could see people doing fun things without me

I watched what seemed like everyone around me keep doing college while I stayed in my room.

In several cases, participants noted feeling responsible for the problems with their existing relationships, echoing the negative self-appraisals we saw in other responses. For instance, when reflecting on their current connections, one participant said those connections were “not reliable, mostly my fault for not investing deeper in more meaningful friendship,” while another expressed “I did not make calling friends and family a priority.” Several participants also noted difficulties with opening up to their support system, which contributed to feeling disconnected from others. One participant described a lack of close others they could open up to: “Not feeling comfortable enough to share my thoughts and feelings with anyone besides my wife due to my developing social skills and lack of opportunity to connect with past friends”; whereas another described a general difficulty with opening up to others: “I have a hard time sharing my feelings with other people.”

#### Desire to Improve Social Connections

In addition to identifying issues with their existing social connections, participants expressed a desire to improve their social connections. Some participants described the desire for increased connection within existing relationships as one of their motivating factors for joining Happify Health. For example, when describing how they were feeling when they joined Happify Health, one participant remarked: “I was wishing that I was more connected to my friends and to my family members.” Another participant similarly commented:

I was wishing to be more connected in that, I wanted what my wife had. In addition to me, she has two close friends she talks to constantly, of whom she has known since high school and in her sorority, respectively. I had a similar experience but failed to maintain those friendships, and I wanted that back or least (sic) be able to cope with my loneliness as I worked towards rebuilding those friendships.

Several participants also expressed a desire to form new relationships. In some cases, this desire was related to changes in circumstances, such as a relocation, that led to a perceived lack of social connection (eg, “I wanted to connect with my local community and new friends which can be hard in a new city”). In other cases, participants expressed wanting connections that were better suited to their needs (eg, “I was looking for alignment and understanding how to bring the right friendships and people into my life to be more happy.”), whereas others noted a genuine lack of connection overall:

I really don’t have that one person I could call just because.

Wish I had a reliable friend who could just cheer me up.

#### Value of Social Connection

Generally, participants saw tremendous value in social connection, even when they felt it was lacking. For example, when asked what role their support system played when they were feeling lonely, one participant remarked: “The biggest role, connecting helps me to not feel lonely anymore.” Another participant who described themself as not having a support system noted: “If I had a support system, I would feel a lot better.” This was particularly evident when people reflected on their recent experiences with social distancing. Some participants felt social distancing measures made the value of social connectedness even clearer.

A sad reality that we’ve taken away the most important element of life - deep, human connection.

We will all value the time we spend together when this is over - and I think that is one positive outlook I’ve gained on social distancing and quarantine.

Others referenced the importance of social connection in helping them cope with the pandemic:

Social connections are very important for helping me cope with this loneliness. During this time, I would crumble(theoretically) without them

In addition, they referenced a sense of comfort from knowing that others are sharing the same experience (eg, “We are helping each other with getting through physical distancing, the struggles and anxiety of unemployment, and reminding one another that we care about each other.”).

### Social Distancing

The final day of the focus group focused explicitly on participants’ experiences during the COVID-19 pandemic. Participants referred to ways in which social distancing measures affected social connectedness as well as other aspects of their life.

#### Impact of Social Distancing on Social Connection

Consistent with preliminary research on the impact of social distancing on loneliness, some participants referred to increased feelings of loneliness and negative affect during this time (eg, “I miss my friends a lot and know how beneficial it was for my health to be able to spend time with them - and I am scared of how I will be able to cope without them.”). However, others referenced positive effects of the pandemic, including opportunities to improve their social connections:

Shelter-in-place has enabled me to dedicate more time to communicate with my support system than I was previously able to while working my normal hours

I interact with more of my friends and family more frequently over zoom and text.

In addition, they referenced a sense of comfort due to the knowledge that most people were feeling the same way:

I would say I don’t feel more lonely now. It’s somewhat comforting to me more people are coping through loneliness and social distancing right now. Where as (sic) before it felt like I was the only one.

#### General Impact of Social Distancing

Participants also described other ways in which the pandemic impacted their lives, ranging from changes in employment and education, the inability to do things they did before, and the impact on their mental health broadly speaking. Given the focus of this paper, we focused primarily on the latter effects. For instance, some participants referenced how restrictions due to the pandemic interfered with their traditional coping mechanisms:

The hardest part is not being able to go to the beach or gym. These two places were my saving grace from everything going on in my life.

In addition, they referenced the restrictions led to other negative consequences like lowered motivation (eg, “Not going in to work and school made me less motivated and productive”). However, again, some participants referred to positive effects, such as offering a respite from their day-to-day lives: “I also feel that social distancing has allowed me to be still and process some of those racing thoughts, while becoming more okay with being by myself.”

#### Use of Technology

Participants also described their increased use of technology for work, school, and social connection as a result of social distancing measures and the COVID-19 pandemic. They often referenced technology as a means of maintaining social connection during the pandemic:

I am coping with loneliness during this time by being on social media alot (sic) more. I have been going to virtual parties and events. I do feel these strategies are helping.

However, they also noted the downsides of technology, particularly *zoom fatigue* (eg, “Zoom meetings are helpful for about an hour, and then I get screen or zoom fatigue and it feels like an obligation because much of my work is via zoom also”). Participants also described that, although technology was necessary for social interaction currently, it was not a replacement for face-to-face interaction: “I’ve learned that digital communication is not a replacement for in-person, but it is significantly better than nothing.”

### Skill Acquisition

During the last 2 days of the focus group, we asked participants about the skills they learned from Happify Health and how they were applying those skills to cope with the pandemic. Notably, although participants were not reminded of the underlying skills promoted within Happify Health, many responses could be classified into our savor, thank, aspire, give, and empathize (STAGE) model. This model reflects categories of activities, each of which emphasize a different skill: *savor* activities focus on mindfulness skills; *thank* activities focus on gratitude; *aspire* activities focus on optimism, goal setting, and finding meaning and purpose; *give* activities focus on kindness, forgiveness, and prosocial behavior; and *empathize* activities focus on self-compassion and perspective-taking.

#### Savoring

Several participants referred to mindfulness, savoring, or meditation when asked to identify two skills they learned from Happify Health. Participants also described using these skills to cope with the pandemic:

Meditation helps with the anxiety. Savoring is useful too. I’m trying to savor the moments that I have inside instead of worrying about what I’m missing on the outside.

In addition, they identified these skills when describing how Happify Health would be helpful in the future: “It can help me in the future with positivity and mindfulness because it is still a strategy I will need to use in my daily life to stay calm and focused.”

#### Thanks

Gratitude was also commonly reported as one of the skills participants learned from Happify Health. Some participants also referenced gratitude activities as those that they found most helpful:

One of the most helpful was sending the letter of appreciation to someone. This exercise was not only gratifying for myself, but for the person I sent it to.

They reference gratitude particularly in terms of helping with their loneliness: “Some of the gratefulness related activities that made me think about people I hadn't talked to in a while or had taken for granted were helpful in dealing with loneliness.”

#### Aspire

Although references to optimism, goal setting, and finding meaning and purpose were less frequently reported as explicit skills participants learned through Happify Health, they made reference to these skills in other responses. Oftentimes these comments were indirect references to optimism (eg, “I do think of positivity as the primary lesson I’ve learned from Happify, even if I can’t remember each individual lesson off the top of my head.”) and goal setting (eg, “It’s given me ideas and motivation to overcome loneliness and made me think of things in a new light”), suggesting perhaps these skills felt less concrete than savoring or gratitude.

#### Give

Direct references to kindness and prosocial behavior were also less commonly reported as explicit skills participants learned from Happify Health but were referenced when participants described the skills from Happify Health they found helpful during the pandemic: “I remember from Happify that saying something nice or doing something nice for others is helpful for me, so I’ve tried to be more encouraging to my friends”; and their overall journey with Happify Health: “I learned how to focus on what I can do for myself and others to brighten my mood on happily (sic).”

#### Empathize

Finally, indirect references to self-compassion were noted as explicit skills learned within Happify Health (eg, patience and self-acceptance) as well as what they found most helpful about Happify Health: “I can’t control loneliness or how people respond to me.” Similar themes appeared when participants described their journey with Happify Health: “I realize that it’s not that I am unloved, it’s that everyone is busy and has trouble reaching out.”; and how they were using skills from Happify Health to cope with the pandemic: “Patience, awareness, breathing, empathy.”

### Coping

The way in which participants discussed their coping strategies appeared to shift across the 3 days, suggesting they implemented the skills referenced earlier to cope with their feelings of loneliness. Over the course of the 3-day focus group, participants referenced using active coping strategies, including strategies that directly or indirectly targeted their loneliness, as well as passive coping strategies. However, participants seemed to reference more active and direct coping strategies, as they reflected on their experiences with loneliness after using Happify Health, compared to day 1, when they reflected on their general experiences with loneliness.

#### Direct Active Coping

Some coping strategies participants described were more problem-focused and directly addressed their feelings of loneliness. One strategy often referenced by participants was making an active effort to connect with friends and family: “I call or text a friend or family member; connecting helps me to not feel lonely anymore.” Notably, however, references to this type of strategy were more frequent when participants were asked to comment on their experiences with loneliness after using Happify Health. Indeed, some participants directly referenced a change in their use of such problem-focused strategies as they reflected on their journey since joining Happify Health. For example, one participant noted:

Happify was the catalyst for really kick-starting my journey towards tackling loneliness. As I worked through the activities, I began to reach out to my friends and rebuild those relationships.

Another participant similarly stated:

I was very busy and disconnected from my friends and family when I started Happify. I am still busy, but I take the time to send a text or make a phone call now. I was a little upset before that people do not reach out to me. But with Happify I was motivated to take the initiative and I have had positive responses.

When reflecting on how Happify Health would help them in the future, another participant noted that the activities would help remind them to proactively reach out to others: “Happify will help me keep connected with people by doing activities where I have to reach out and interact with them.”

#### Indirect Active Coping

Participants also described more indirect strategies to actively address their loneliness. These strategies involved methods to generally improve mental health while not necessarily directly targeting loneliness or social connections, such as practicing mindfulness or self-care. For example, when reflecting on how they cope with loneliness in general on day 1, one participant noted:

I cope with loneliness by finding and doing things that I enjoy when alone. I watch my favorite shows, make my favorite beverages, and do my favorite activities like writing, doodling, listening to music, cleaning, or self-care.

Another participant indicated:

talking instead of bottling up my feelings helped. I also did yoga and got a physical outlet for my anger.

As with the more direct techniques, indirect coping strategies were mentioned more frequently, and with increased contemplative detail, in the latter 2 days of the focus group, when participants reflected on their experiences since joining Happify Health. When one participant described their journey with Happify Health, they noted the following:

I have learned to breathe. This is something I often never really do with intention. I have also learned to slow down and accept the things I can’t change. Others are not judging me or worrying about the things I am. It’s all in my head.

Another stated: “I have learned not to take everything so seriously and stay in the present moment when needed.” Some participants also referred to these indirect coping strategies when asked how Happify Health could help them in the future. For example, one participant noted the benefits of Happify Health in the future were:

meditation guidance, relaxation, etc. I feel like I would use Happify as a standard relaxation app

Similarly, when asked to reflect on how skills they learned by using Happify Health have helped them cope with the pandemic, one participant responded:

Meditation has been huge for me right now. It brings me back to my center and keeps me out of my head. I’ve been practicing every morning to start my day and it really helps.

Another stated:

I learned to knock out negative thoughts by refocusing on the positive. I have learned to slow down and meditate when I am anxious.

#### Passive Coping

Participants also shared passive coping techniques they used to manage feelings of loneliness. Passive strategies included watching television, listening to music and podcasts, staying busy with tasks and activities, etc. For example, one participant described their methods for coping with loneliness as:

cooking, listening to music, medication, reading, and zoning out in front of the TV. I don’t feel as lonely when I hear music or TV voices in the background.

Another shared:

Staying busy and focusing on completing all the things I need to get done will help me manage my loneliness. When I set my mind to something I can usually only focus on that and keep the bad lonely thoughts at bay

Importantly, in comparison to active strategies, the motivation behind these actions did not appear to address the underlying issues with loneliness or improve mental health in general. Rather, these strategies reflected more temporary attempts to distract oneself from the negative feelings associated with loneliness.

Although these strategies were referenced more frequently when participants reflected on their experiences prior to using Happify Health, passive strategies were still mentioned as coping techniques following their use of Happify Health as well. In fact, some participants referenced using the platform itself as a form of distraction. One participant described the games within the platform having “distracted from the negative feeling and provided a replacement activity,” whereas another noted “I think it could be a good distraction for me” when asked how Happify Health could help them in the future.

## Discussion

Loneliness has been identified as a growing public health concern due to its increasing prevalence [3] and association with other mental and physical health problems [9-13]. However, surprisingly little research has explored the impact of interventions for loneliness, particularly digital interventions. Moreover, much of the existing literature has focused on older adults, and consequently, we know less about the experience of loneliness for younger adults or their reactions to digital interventions targeting loneliness. The goal of this study was to explore how self-identified lonely adults aged 18-64 years experience loneliness and their experience with a digital mental health intervention using a qualitative, asynchronous focus group design.

### Experience With Social Distancing

Although the primary aim of this study was to explore adults’ experiences with loneliness and reactions to the Happify Health platform, the focus group occurred amid the COVID-19 pandemic when most of the United States was under stay-at-home orders. Consequently, a secondary aim of the study was to explore participants’ experiences with loneliness during the pandemic. This was of particular interest because researchers have expressed concern that social distancing measures in place to prevent the spread of COVID-19 may result in widespread increased loneliness [22].

Some participants in this study did reference feeling more loneliness and negative effects as a result of social distancing measures; however, some participants also described positive effects of the pandemic and social distancing measures. Positive effects included more opportunities for social contact (often facilitated by technology), a sense of comfort resulting from the fact that everyone was facing similar circumstances, and respite from one’s day-to-day life. Although references to these positive effects might be surprising given the focus on the potential negative effects of social distancing and some preliminary evidence suggesting increases in loneliness [24], other research also suggests that loneliness may not have increased as much as people feared during the beginning of the COVID-19 pandemic [63]. For instance, one nationwide study assessed US residents at 3 different periods of time: before the COVID-19 outbreak in the United States, in late March 2020, and in late April 2020 (the same time frame when this focus group was conducted). They found no significant changes in average levels of loneliness over time. Although older adults specifically showed an increase in loneliness during the acute phase of COVID-19, loneliness actually decreased following the implementation of stay-at-home orders, when social distancing measures would have been the most strict [64]. In fact, participants reported an increase in perceived social support from the first assessment (before the COVID-19 outbreak) to subsequent assessments, with no significant change in perceived social support from late March to late April 2020 [64]. Another study found less than a 3% increase in the prevalence of loneliness between 2018 and during the pandemic in 2020 [65]. In other words, the effect of the COVID-19 pandemic on loneliness is not as clear as researchers originally predicted.

One potential explanation for this is that we underestimated the impact of digital technology during this crisis. Digital technology can be leveraged to create opportunities for social connection [22,66], helping to curb feelings of loneliness. Indeed, social networking apps like *Facetime* and *Skype* were recommended to older adults during the COVID-19 pandemic [67] because research suggests these apps help to reduce their risk of depression [68]. Video chat platforms also increased in popularity during the pandemic [69], which might help to explain the increase in perceived social support early on. Participants in this study also often referenced using technology to maintain social connection during the pandemic, including social media, text messaging, and video chat. Some even referenced being more connected than before because of their use of these technologies.

However, it is important to note that the focus group was conducted just 2 months into the pandemic, and much of the published research on trends in loneliness during the pandemic similarly reflect early responses to the COVID-19 crisis and social distancing measures. Given that participants also referenced fatigue related to the increased use of technology and that virtual communication did not replace face-to-face interaction, it is conceivable that communication patterns have changed over time. If increased virtual communication was a temporary reaction to stay-at-home orders and was not maintained over time, it is possible that the predicted increase in loneliness associated with the pandemic was merely delayed. Thus, more research is needed to better understand how trends in virtual communication and loneliness may have changed throughout the COVID-19 pandemic.

### Experience With Loneliness

Researchers have defined loneliness as a feeling stemming from the perception that one’s social network is insufficient or unsatisfactory [1]. Contrary to popular belief, these feelings do not necessarily result from social isolation but rather from perceptions that one’s expectations are not being met [70]. Consequently, loneliness is a subjective state—although two people may experience similar circumstances, like relocating often, one may feel lonely while the other does not [71].

Although our sample consisted only of individuals who indicated they desired feeling more connected to others when signing up for Happify Health, the subjective nature of loneliness was evidenced in the heterogeneity of participants’ descriptions of their relationships and the causes for their loneliness. Participants did predominantly reference negative perceptions of their existing social network; however, some participants felt they lacked a support system altogether, whereas others clearly identified close others but felt disconnected from their support system. Furthermore, among those who felt more disconnected from their support system, there was variability in their reasons for feeling disconnected, including feeling “different” from close others, feeling excluded or ignored by close others, or feeling as though they were the ones responsible for the lack of intimacy and contact.

Similarly, participants differed in how they described their experiences with, or sources of, loneliness. Some participants attributed their feelings of loneliness to more proximate, circumstantial triggers. Others, however, described a chronic pattern of loneliness, sometimes coupled with other mental health concerns like social anxiety.

The heterogeneity in participants’ experiences with loneliness could have important implications for treatment. Although research suggests that loneliness interventions that incorporate social cognitive training are more effective than other types of loneliness interventions, most loneliness interventions continue to focus primarily on increasing opportunities for social interaction [31]. Conceivably, individuals who lack a support system altogether or who are experiencing temporary loneliness due to a change in circumstances may benefit from behavioral interventions that focus on social skills training and increased social interaction to build a support system. However, individuals whose loneliness stems from feeling misunderstood or excluded by their social network, or those with chronic patterns of loneliness, may require interventions with more of a cognitive component to address maladaptive cognitions that may contribute to their perceptions of their existing support system. Indeed, participants’ responses throughout the focus group suggested that participants differed in their motivation to address their loneliness and whether their primary goal was to create new social connections or to improve existing connections. Thus, to be effective, loneliness interventions may need to draw on a variety of strategies, not just increasing opportunities for social contact, and allow for personalization to directly address the individual’s unique experience with loneliness.

### Negative Self-perceptions

Another common theme in participants’ responses throughout the focus group was negative self-appraisal. Consistent with research suggesting loneliness and low self-esteem have a reciprocal relationship [72], the ways in which participants referred to negative self-appraisals included both causes for and consequences of their loneliness. For example, in some cases, participants attributed the problems with their relationships to themselves, whether it was due to a lack of self-disclosure to close others or because they did not invest enough energy into their relationships. However, participants also described a process whereby feeling lonely led to questions about their self-worth. For instance, some participants described how they interpreted the lack of social contact as evidence that they were annoying or worthless. For some participants, the negative self-appraisals even reflected more sweeping feelings of self-disgust (eg, “I haven’t done anything today and I’m all by myself - what a waste of space.”), a variable that has become increasingly important in relation to loneliness and appears to mediate the relationship between loneliness and depression [73,74].

These data point to the importance of incorporating strategies for addressing underlying negative cognitions about the self when treating loneliness. Indeed, in a recent paper, Ypsilanti [75] argued that loneliness interventions should address feelings of self-disgust that often coincide with loneliness, which may contribute to social avoidance and maintain loneliness. Thus, interventions focusing primarily on increasing opportunities for social contact may not be effective without addressing the negative self-appraisals that may interfere with forming social connections.

### Experience With the Digital Intervention

Given the lack of research on loneliness interventions, particularly digital interventions, another goal of this study was to better understand participants’ experiences with the Happify Health platform. The pilot study we recruited participants from provided preliminary evidence that completing more activities on the platform may help to reduce feelings of loneliness among lonely adults between 18-64 years of age [56]. However, the aim of this focus group was to obtain more in-depth information about participants’ reactions to the intervention, including the extent to which they adopted new skills and coping strategies as a result of completing the program.

Notably, Happify Health is a not a specific loneliness intervention and not all participants were drawn to the program because of their loneliness. Given the aforementioned argument that interventions may need to be flexible and individualized because of the subjective nature of loneliness, offering people struggling with loneliness choice in interventions may be important in the same way person-activity fit influences the impact of interventions [76]. Some participants appeared to prefer addressing their loneliness more indirectly and even as part of a broader program addressing other issues, as evidenced by the fact that not all participants started or completed the recommended loneliness intervention. However, other participants appeared to enjoy the prospect of tackling their loneliness directly, as illustrated by the fact that some participants who did not even label themselves as “lonely” described feeling empowered and motivated when the loneliness intervention was recommended to them.

In addition, participants showed evidence of learning various skills taught via Happify Health activities and, more importantly, implementing those skills to use more active coping strategies for loneliness. That is, there was a noticeable change in how participants described their coping mechanisms when asked to reflect on their experiences with loneliness prior to using the Happify Health platform compared to the latter 2 days of the focus group, when they were asked to reflect on their experiences with Happify Health and during the pandemic. Although references to passive coping strategies did not appear to change over the course of the focus group, participants referred to more active coping strategies when reflecting on their experiences since joining Happify Health. Moreover, their descriptions of these coping strategies felt richer and more intentional. For example, participants were able to explain why they used a particular skill or coping strategy and how it helped with their loneliness. Although this study cannot speak to whether these participants were able to reduce their loneliness by using such coping strategies, these data suggest participants felt they gained both direct (eg, actively reaching out to others) and indirect (eg, mindfulness exercises) strategies they could use when feeling lonely as a result of completing the intervention.

Interestingly, we found that the two skills participants commonly referred to explicitly were mindfulness skills and gratitude. Recall that most loneliness interventions focus on increasing opportunities for social contact [31]; however, neither of these skills directly focus on social contact or social skills. Nevertheless, participants described how these exercises helped them with their loneliness and how they would continue to use them in the future. This further points to the importance of moving beyond the notion that social skills training and increasing opportunities for social contact are sufficient for addressing loneliness. Indeed, there is a growing body of literature that building mindfulness skills can help to reduce loneliness and even increase social contact [48,77]. Although the mechanism by which mindfulness reduces loneliness is unclear, participants in this study described how savoring and focusing on the present helped to reduce maladaptive cognitive patterns associated with loneliness that elicited anxiety and negative self-concepts. Similarly, cross-sectional research suggests gratitude and loneliness may be inversely related [78,79]. Again, although the reasons for this inverse relationship are unclear, participants described how practicing gratitude, particularly when it involved expressing gratitude to a close other, helped to reframe their perceptions of existing social connections and introduced opportunities for social contact, all while promoting positive self-appraisals and affect.

Interestingly, some participants also described completing Happify Health activities as a means of distracting themselves when they felt lonely. In other words, the program itself acted as a passive coping strategy. However, unlike other passive coping strategies participants referenced, like “zoning out in front of the TV,” users also gained helpful skills while distracting themselves. This points to yet another potential benefit of digital loneliness interventions: they are available on demand, offering lonely people a productive means of distracting themselves when they may not want to actively and directly address their loneliness.

### Limitations and Future Directions

This study provides rich information about participants’ experiences with loneliness and with a digital mental health platform; however, there are limitations to the design that may reduce the generalizability of these findings. First, although we asked participants about their general experiences with loneliness and noted changes in how participants described coping strategies when describing their experiences before and after using the Happify Health platform, all of the participants’ responses were collected after they had used the Happify Health platform for 8 weeks or longer. Consequently, it is plausible that their reflections on their experiences with loneliness before using the digital intervention may be biased based on their experiences with the platform or their current level of loneliness. Similarly, although we were able to collect valuable information about participants’ experiences with loneliness during the COVID-19 pandemic, the fact that we conducted the focus group during the early stages of the pandemic and when most of the United States was under stay-at-home orders, it is also plausible that participants’ current experiences influenced their reflection on their general experience with loneliness as well as their experience with Happify Health. In addition, although we explored participants’ reactions to the Happify Health program, including their reasons for joining Happify Health and their thoughts on the activities and tracks they completed, we did not inquire about negative side effects. Although participants did not spontaneously refer to negative side effects while using the platform in their responses, direct questions asking participants if they had negative side effects or reactions while using the platform should be included in future research to better assess participants’ experiences.

Another limitation is potential selection bias that reduces the representativeness of the focus group. As with many qualitative studies, this group consisted of a small number of participants that may not be representative of the general population. Due to a technical issue, we were unable to link participants’ responses in the focus group to their demographics and questionnaire responses from the pilot study, making it difficult to determine the extent to which our focus group was diverse in terms of age, level of loneliness, geographical location, etc.

In addition to the small sample, participants were invited to participate in this study if they had participated in the preceding pilot study and were assigned to the active intervention, completed at least 16 activities on Happify Health during that study, and completed the pre- and posttest assessments for the pilot study. Consequently, the reflections on loneliness and the digital intervention we coded may only represent the experiences of successful Happify Health users, and the experience of those who completed fewer intervention activities or stopped using the program altogether remain unclear. Moreover, given the small number of participants overall, the insights drawn from participants’ responses should be considered preliminary, as different themes may have emerged with a larger sample of participants.

Considering these limitations, it will be important to explore these themes with a larger, more representative sample. In particular, a study in which participants reflect on their experiences with loneliness before they start using the platform and then again after using the platform would help to reduce the potential bias of retrospective reports.

### Conclusions

Loneliness is a growing public health concern, perhaps even more so given the recent global pandemic that resulted in widespread social distancing measures. However, interventions targeting loneliness are limited and often focus primarily on increasing opportunities for social interaction and improving social skills. Meanwhile, a growing body of literature points to the importance of cognitive-behavioral techniques, mindfulness, and gratitude for effectively addressing loneliness. Although more research with a larger and more representative sample is needed, the responses we reviewed from adults aged 18-64 years who self-identified as lonely over the course of a 3-day focus group can be summarized into three broad recommendations for loneliness interventions.

First, given the subjective and complex nature of loneliness, loneliness interventions should incorporate a variety of techniques to address loneliness, and these should include both direct (eg, social skills training and cognitive behavioral techniques) and indirect (eg, mindfulness and gratitude) strategies to accommodate different levels of motivation for addressing loneliness. Second, given the role of negative self-appraisals and self-disgust as a cause and consequence of loneliness, to be effective, loneliness interventions need to incorporate strategies to address these underlying beliefs as well. Finally, digital interventions may be particularly useful for addressing loneliness, as they may provide a convenient but productive option for passive distraction when someone is feeling lonely.

## Appendix

Multimedia Appendix 1 Full list of questions and activities by focus group day.

## Abbreviations

RCT: randomized controlled trial
STAGE: savor, thank, aspire, give, and empathize
