# Supplementary material for: The Impact of a Digital Intervention (Happify) on Loneliness During COVID-19: Qualitative Focus Group
Source: JMIR Ment Health. 2021 Feb 8;8(2):e26617. doi: 10.2196/26617 (PMC7872202; doi:10.2196/26617)
Supplement: Multimedia Appendix 1 [file mental_v8i2e26617_app1.docx]

**Supplemental Material**

**Table.** Full list of Questions and Activities by Focus Group Day.

| **Activity** | **Question** |
| --- | --- |
| **Days 1-2: Welcome** | |
| *Introduce Yourself* | “To begin, please introduce yourself and tell us a little about you and your support system. Then, post an image that illustrates how you feel today and tell us about it. You can use your own photo or find something online, but please do not post a picture that includes the faces of you or individuals you know.” (open-ended) |
| **Day 3: Understanding Loneliness** | |
| Activity 1: *Feeling Connected* | “When you first joined Happify, you said that you wished you were more connected to others. Reflecting back, can you tell me about what was going on in your life at the time? In what way(s) were you wishing to be more connected? How were you feeling?” (open-ended) |
| Activity 2: *Your Experience with Loneliness* | “What words would you use to describe yourself when you feel lonely?” (open-ended) |
|  | “In your experience, what makes you feel lonely?” (open-ended) |
|  | “On a scale from 1 (*not at all*) to 5 (*extremely*), how much is loneliness a concern for you?” (close-ended) |
|  | “What do you do when you feel lonely? How do you cope” (open-ended) |
|  | “What role does your support system play when you feel lonely?” (open-ended) |
| Activity 3: *Thinking - Feeling - Saying - Doing* | “Can you tell me about the last time you felt lonely and why? When was this?” (open-ended) |
|  | “What were you thinking at this time? Can you describe at least three thoughts you had?” (open-ended) |
|  | “What were you feeling at this time? Can you describe at least three feelings you had?” (open-ended) |
|  | “What were you saying to others at this time? Can you describe the interactions you had with your support system during this time?” (open-ended) |
|  | “Finally, what types of things were you doing to cope at this time? What helped?” (open-ended) |
| **Day 4: Using Happify** | |
| Activity 1: *Your Experience Joining Happify* | “What made you want to join Happify? What were you hoping to achieve? (open-ended)” |
|  | “When Happify recommended the “Defeat Loneliness” track what did you think? Why?” (open-ended) |
|  | “After being presented the “Defeat Loneliness” track recommendation did you:” (close-ended; response options: “start and complete the track”, “start the track but switch to a different track partway through”, “choose a different track from the start”) |
|  | “Tell me more about how and why you made your track choice. If you chose to switch or chose a different track from the start, what other track did you choose and why?” (open-ended) |
|  | “If you completed multiple tracks, what other tracks did you complete? Why did you choose them?” (open-ended) |
|  | “What did you think about the activities you were asked to complete? Which ones were most and least helpful? Why?” (open-ended) |
| Activity 2: *Your Happify Journey* | “For this task I’d like you to reflect on your journey over the past few months, from the time you started using Happify to today. Tell me about your experience with loneliness during this time. How has Happify played a role in this experience?” (open-ended) |
| Activity 3: *Your Thoughts on Happify* | “Two skills I learned on Happify were ______ and _______” (fill-in-the-blank) |
|  | “In the context of feeling lonely, the most helpful part of Happify was _______ because ________.” (fill-in-the-blank) |
|  | “Two things that would make Happify better for people who feel lonely would be ______ and ______.” (fill-in-the-blank) |
|  | “My overall experience using Happify was _______ because _______.” (fill-in-the-blank) |
| **Day 5: Your Experience with Social Distancing** | |
| Activity 1: *Social Distancing* | “To start, can you tell me what social distancing means to you?” (open-ended) |
|  | “How has social distancing changed your day-to-day life in the past month?” (open-ended) |
|  | “What words would you use to describe how social distancing makes you feel? Why?” (open-ended) |
|  | “On a scale of 1 (*not at all*) to 5 (*extremely*), to what extent has social distancing made you feel more lonely?” (close-ended) |
|  | “Please describe how social distancing has or has not made you feel more lonely. Why?” (open-ended) |
|  | “What is the hardest part of social distancing for you? Why?” (open-ended) |
| Activity 2: *Coping and Staying Connected* | “How are you coping with loneliness during this time?” (open-ended) |
|  | “Do you feel these strategies are helping? Why or why not?” (open-ended) |
|  | “If you haven’t mentioned this already, are you staying connected with others during this time? Who are you staying connected to? Why? How?” (open-ended) |
|  | “Are social connections helpful for you in coping with loneliness during this time? Why or why not?” (open-ended) |
|  | “On a scale of 1 (*not at all*) to 5 (*extremely*), to what extent is staying connected with others an important part of coping with loneliness for you?” (close-ended) |
|  | “Reflecting on Happify, what skills have you learned that are helping you cope at this time?” (open-ended) |
| Activity 3: *Thinking Towards the Future* | “What words would you use to describe how you are feeling about the next few months? Why?” (open-ended) |
|  | “On a scale of 1 (*not at all*) to 5 (*extremely*), when thinking about the next few months, how much is loneliness a concern for you?” (close-ended) |
|  | “Please describe why or why not.” (open-ended) |
|  | “What skills or lessons have you learned over the past month that will be important to maintain in the future when managing loneliness? Why?” (open-ended) |
|  | “When the coronavirus pandemic is over, and things return to normal, do you plan to make any long-term changes in your life to manage feelings of loneliness? If so, what? If not, why?” (open-ended) |
|  | “How do you think Happify can help you in the future? Why?” (open-ended) |
|  | “Considering all of the things we have discussed over the past few days, what else do you think we should know about your experience with loneliness in general and in the context of social distancing?” (open-ended) |
